# Histone Acetyltransferase Rtt109 Regulates Development, Morphogenesis, and Citrinin Biosynthesis in *Monascus purpureus*

**DOI:** 10.3390/jof9050530

**Published:** 2023-04-29

**Authors:** Ruoyu Shi, Pengfei Gong, Qiaoqiao Luo, Wei Chen, Chengtao Wang

**Affiliations:** 1 Key Laboratory of Geriatric Nutrition and Health, Ministry of Education, Beijing Advanced Innovation Center for Food Nutrition and Human Health, Beijing Engineering and Technology Research Center of Food Additives, Beijing Technology & Business University (BTBU), Beijing 100048, China; 2 Yunnan Plateau Characteristic Agricultural Industry Research Institute, Yunnan Agricultural University, Kunming 650201, China

**Keywords:** histone acetyltransferases, secondary metabolism, *Monascus*, edible fungi, CRISPR/Cas9

## Abstract

Histone acetyltransferase (HAT) has been reported to be pivotal for various physiological processes in many fungi. However, the functions that HAT Rtt109 perform in edible fungi *Monascus* and the underlying mechanism remains unclear. Here, we identified the *rtt109* gene in *Monascus*, constructed the *rtt109* knockout strain (Δ*rtt109*) and its complementary strain (Δ*rtt109*:com) by CRISPR/Cas9 methods, and functionally characterized the roles that Rtt109 play in *Monascus*. Deletion of *rtt109* significantly reduced conidia formation and colony growth, whereas, it increased the yield of *Monascus* pigments (MPs) and citrinin (CTN). Further real-time quantitative PCR (RT-qPCR) analysis indicated that Rtt109 remarkably affected the transcriptional expression of key genes related to development, morphogenesis, and secondary metabolism of *Monascus*. Together, our results revealed the critical roles of HAT Rtt109 in *Monascus*, and enriched our current knowledge of the development and regulation of secondary metabolism in fungi, throwing light on restraining or eliminating citrinin in the development and industrial applications of *Monascus*.

## 1. Introduction

Histone post-translational modifications play an important role in regulating transcription [1]. These processes include methylation, acetylation, phosphorylation, sulfonylation, and ubiquitination [2]. Acetylation of histones is a reversible and dynamic process that is mediated by histone acetyltransferases (HATs) and histone deacetylases (HDACs). These two histone-modifying enzymes have opposite activities and interact with each other [3]. HATs transfer acetyl groups from acetyl coenzyme A to cofactors with lysine residues and transform the chromatin structure to activate transcription. Conversely, HDACs catalyze the removal of this modification, thereby inhibiting transcription [4]. These enzymes are responsible for the reversible dynamic process of acetylation and are required for chromatin remodeling and transcriptional regulation [5,6].

Studies have shown that many cellular physiological processes in fungi are controlled by the acetylation of histone residues [7]. HATs and HDACs are involved in regulating fungal development, adaptation to environmental stress, and virulence. For example, Gcn5, a KAT2 family HAT, plays an important role in chromatin remodeling of the promoter, promotes transcriptional activation in fungi, stress tolerance, and regulates epigenetic inheritance [8,9,10]. Rtt109 is an acetyltransferase that acetylates histone H3, before binding to DNA at K56 and N-terminal residues [11]. Rtt109 is a type of HAT that acetylates histone H3 lysine 56 (H3K56), which is required for DNA replication-coupled nucleosome assembly and genome stability [12]. In fungi, Rtt109 has been identified as a fungal-specific KAT11 family histone acetyltransferase, which is mainly responsible for the acetylation of histone 3 lysine 56 (H3K56) [13]. Many acetyltransferases, including Rtt109, have been reported in fungal species, such as *Saccharomyces cerevisiae*, *Aspergillus fumigatus*, and other fungi, and in the bacterium *Staphylococcus* [14,15,16]. Rtt109, in *S. cerevisiae*, is responsible for H3K56 H3K9, K14, K23, and K27 acetylation [17]. Rtt109 is critical for the acetylation of histone H3 lysine 56 sites to preserve genomic integrity and pathogenicity in *Candida albicans*. Rtt109 activity is triggered through an unknown method, by attaching two distinct histone chaperones, Vps75 and Asf1 [18]. Deletion of *rtt109* or *asf1* in yeast cells eliminates the acetylation of H3K56 and impairs DNA repair [19,20]. In *Aspergillus flavus*, Rtt109 regulates growth, conidia formation, nucleus formation, toxin synthesis, environmental stress response, and infestation [21]. The Δ*rtt109* mutant causes *A. flavus* to lose the formation of asexual spores, affects sclerotia synthesis, and downregulates the expression of genes related to the biosynthesis of *brlA* and *abaA*. Aflatoxin synthesis in the *A. flavus* ΔRtt109 strain is decreased. Rtt109 deficiency leads to severe defects in trophic growth and conidiation, reduced virulence, and hypersensitivity to genotoxic agents in *A. fumigatus* [22].

*Monascus* is a traditional edible filamentous fungus that has been used for thousands of years in China, and is widely used in medicine, food, and industry. *Monascus* produces various natural and functional secondary metabolites, such as *Monascus* pigments (MPs), monacolin K (MK), and *γ*-aminobutyric acid (GABA) [23]. MPs are widely used as natural pigments in food, whereas MK lowers serum cholesterol levels [24]. However, during fermentation, *Monascus* produces small amounts of citrinin (CTN), a mycotoxin that causes kidney and liver damage, seriously hindering the development of the *Monascus* industry [25].

In filamentous fungi, histone acetylation has been shown to affect fungal growth, development, and secondary metabolism. Some epigenetic factors, such as methylation, acetylation, and phosphorylation, affect the growth and development of filamentous fungi and the synthesis of *Aspergillus* toxins. The histone H3K4 methyltransferase complex *Ash2* is critical for mycelial development and secondary metabolism. *Ash2* is involved in spore germination, pigment production, and CTN production, and plays a controlling role in purple spore development and secondary metabolism in *M. purpureus* [26]. HAT MrGcn5 regulates the production of CTN in *M. ruber*; deletion of *MrGcn5* results in lower CTN production, and *MrGcn5* supplementation recovers the CTN level [27]. The HDAC MrRpd3 also regulates the biosynthesis of CTN in *Monascus*, and overexpression of *Mrrpd3* significantly promotes CTN yield in *M. ruber* [28]. 

However, the function of HAT Rtt109 has not been reported in *Monascus*, and whether Rtt109 affects the growth, morphology, and secondary metabolism in *Monascus* is still unknown. LaeA is a global regulatory factor for secondary metabolites found in *Aspergillus nestoris* [29], which can regulate the generation of secondary metabolites, as well as the growth of mycelium and morphological differentiation of the conidia [30]. It has been reported that overexpression of LaeA can activate MK gene cluster of *Monascus* [31]. VeA is another global regulator, which, together with VelB, VosA, and LaeA, regulate morphological differentiation and secondary metabolism. Disruption of *veA* significantly reduced CTN production. The developmental regulator WetA and VosA are critical for conidial maturation in *Beauveria bassiana*, and lack of the two genes resulted in an almost total depression of two central development activators, *brlA* and *abaA* [32]. In this study, we evaluated the effect of Rtt109 on the development, morphogenesis, MPs, and CTN biosynthesis in *Monascus purpureus* M1, *rtt109* knockout strain Δ*rtt109*, and the complementary strain, Δ*rtt109*:com. Through the analysis of the transcriptional level of regulatory genes in MPs and CTN synthesis, conidial development control genes *brlA*, *wetA*, and global regulatory gene *laeA*, we found that HAT Rtt109 was involved in key processes, such as filamentous growth and development, conidia formation, spore wall assembly, and secondary metabolites biosynthesis.

## 2. Materials and Methods

### 2.1. Gene Sequence and Phylogenetic Tree Analysis

Sequence and phylogenetic tree analysis were performed to obtain the Rtt109 protein sequence of *M. purpureus*. BLASTp was performed to search for protein homologues of the *Aspergillus fumigatus* Rtt109 protein (GenBank: EDP51698.1); thus, interesting sequences were downloaded from the NCBI (National Center for Biotechnology Information resource). These sequences were compared by MEGA 6 software (Mega Limited, Auckland, New Zealand) and CLUSTALW (https://www.genome.jp/tools-bin/clustalw, accessed on 11 November 2022). Mapping of sequence alignment was performed by using ENDscript/ESPript (http://espript.ibcp.fr/ESPript/cgi-bin/ESPript.cgi, accessed on 11 November 2022). Predicted protein domains were evaluated using the SMART interface (http://smart.emblheidelberg.de/, accessed on 11 November 2022). The structural domains of each protein were mapped by IBS software.

### 2.2. Strains, Media, and Cultural Conditions

*M. purpureus* strain M1 (CGMCC 3.0568) was taken as the wild-type (WT) control strain. All strains were maintained on potato dextrose agar (PDA) medium at 30 °C for mycelium collection. The PDA with 1.2M sorbitol and 20 μg/mL hygromycin B (Sigma-Aldrich, Shanghai, China) was used for protoplast regeneration and transformation resistance screening. Ampicillin (100 μg/mL) was supplemented when required. For phenotypic characterization and colonial observation, 4 different types of media were used: PDA, malt extract agar (MA), 25% glycerol nitrate agar (G25N) media, and Czapek yeast extract agar (CYA). An amount of 10^6^/mL fresh spores were inoculated into 50 mL PDB medium, and were continuously shaken at a speed of 120 rpm at 28 °C. The strains were inoculated into different media at 28 °C, respectively. After 7 days of growth, the morphology was observed, and the ascospore and conidiospore of strains were counted [28]. *Escherichia coli* DH-5α was cultured in an LB medium as a host for the conventional plasmid subclone.

To determine the yield of secondary metabolites, liquid and solid fermentation were conducted, respectively. The strains were cultured in fermentation broth at 25 °C, 150 rpm, for 15 days [33]. For citrinin detection, 2 mL of fresh spore solution (10^6^ spores/mL) was taken and inoculated into 30 g of rice, and cultured at 28 °C [27]. From the 3rd day to the 15th day, fermented rice and fermentation broth were collected every three days. Fermented rice was dried and ground into powder, and the output of pigment and citrinin was analyzed.

### 2.3. Construction of Plasmids and Mutant Strains

The plasmids and primers used in this study are listed in Table S1. The construction of *rtt109* knockout plasmids was performed, as described by Liu et al. [30]. The genomic DNA of *M. purpureus* was extracted using the DNA Extraction Kit (Tiangen, Beijing, China), according to the manufacturer’s instructions. Based on CRISPR/Cas9 methods, the homology arms were amplified with primer pairs *rtt109*-up1000-F/*rtt109*-up1000-D and *rtt109*-up1000-F/*rtt109*-up1000-D from the genomic DNA. The vector fragment was amplified using plasmid pUC57 as the template, with primers pUC57-F/pUC57-R. Purified fragments were assembled using the Hieff Clone Plus Multi One Step Cloning Kit (YEASEN, Shanghai, China), resulting in plasmid KL-03(*rtt109*). Primers of pFC332-rtt109-sgRNA-DNA1-F/R and pFC332-rtt109-sgRNA-DNA2-F/R were used to amplify 6 bp-reverse-complementing sequences and 20 bp-sgRNA genes. The vector fragment was amplified using plasmid pFC332 as the template, with primers pFC332-F/ pFC332-R. Purified fragments were assembled using the Gibson assembly method, resulting in plasmid KL-05(*rtt109*), which were inserted into BglII/PacI-digested pFC332 to generate pKL-05.

The primers *rtt109*-F/R were used to amplify the *rtt109* gene, with the genomic DNA as the template. The vector fragment was amplified with pBARGPE1-F/R, using plasmid pBARGPE1-*hygro* (Miaoling, Wuhan, China) as the template. Purified fragments were assembled using the Gibson assembly method, resulting in plasmid pBARGEP-*hygro*-*rtt109*. 

The construction of the *rtt109* deletion strain (Δ*rtt109*) was performed, as described by Liu et al. [34]. When the *rtt109* deletion strain (Δ*rtt109*) was obtained, the construction of *rtt109* complemented strain (Δ*rtt109*:com) was performed, as described by Zhang et al. [35]. The pBARGEP-*hygro*-*rtt109* plasmid was incubated with 100 μL of *M. purpureus* Δ*rtt109* strain protoplasts to construct the complementary strain Δ*rtt109*:com. The deletion and complementation of *rtt109* gene were verified by diagnostic PCR [34]. The genomic DNA extracted from the transformant colonies were used as templates of diagnostic PCR. 

### 2.4. Detection of Monascus Pigments and Citrinin Production 

An amount of 3 mL of 70% ethanol solution was added to 1 mL of *Monascus* fermentation broth, followed by 60 min of water bath at 60 °C [35]. The absorbance values at 505 nm, 448 nm, and 410 nm were measured, and the color values of red pigment, orange pigment, and yellow pigment were calculated according to the formula. The absorbance values were used to express the pigment content. The color values of samples were obtained by OD_505_, OD_448_, and OD_410_, by multiplying the dilution ratios, respectively [36].

The method for extracting citrinin is according to the method of Ouyang, Liu, Wang, Huang, & Li [37], with modification. The citrinin concentration of fermentation broth was determined by the enzyme-linked immunosorbent assay (ELISA) kit (MEIMIAN, China). The fermented rice was dried and ground into powder, 20 mg with 1 mL 80% methanol was extracted, and the content of citrinin was detected by UPLC (Agilent 1290, Germany) and the method of [38]. The mobile phase is solvent A (0.1% formic acid aqueous solution) and solvent B (acetonitrile), 0.1% formic acid: acetonitrile (*v*/*v*) = 1:1, the flow rate is 1 mL/min, and the injection volume is 2 μL. Detect with a fluorescence detector at 330 nm, and keep the column temperature at 30 °C.

### 2.5. Phenotypic Assays, Morphological Mycelium Observation 

To perform phenotypic analysis to assess colony morphology and mycelial growth, the conidia suspension (2 μL, 1 × 10^6^/mL) was inoculated on four solid mediums (PDA, MA, G25N, CYA). The conidia suspension was added to PDB and shaken at 200 rpm for 7 days. Mycelia were collected and dried, and the dry weight was recorded as dry cell mass. To determine the production of conidia, count the number of conidia with a hemacytometer after incubation at 37 °C for 48 h. These strains were maintained on PDA at 30 °C for 7 days, and the number of spores was calculated with a hemocytometer, according to the previous description [39].

The micromorphology of the strains was examined using the scanning electron microscope (SEM, Su8020, Hitachi Ltd., Tokyo, Japan). After 3 days of liquid fermentation, the mycelium was fixed for 12 h in a solution containing 2.5% glutaraldehyde. Mycelial was then rinsed twice with phosphate-buffered saline (PBS, pH 7.2). Dehydration was carried out with various ethanol concentrations (30%, 50%, 70%, 80%, 90%, 100%), with two repeats of each concentration and a dehydration period of 10 min. The supernatant was removed each time by centrifugation at 12,000 rpm for 10 min. Mycelia were suspended in a 1:1 mixture of isoamyl acetate and ethanol, before being put in an isoamyl acetate solution. After discarding the supernatant, the samples were dried at 60 °C in hexamethyldiazolidine (HMDS) solvent.

### 2.6. RNA Isolation and Real-Time Quantitative PCR (RT-qPCR)

A RT-qPCR was performed to assess the degree of expression of associated genes. Total RNA was extracted from mycelium grown in PDB medium by the RNAprep Pure Plant Kit (Tiangen-bio, Beijing, China). The total RNA was reverse transcribed, following the directions of a first-strand cDNA reverse transcription kit (Fast Quant RT Ki, Tiangen, Beijing, China). The SuperReal PreMix Plus was used for the RT-qPCR (SYBR Green, Tiangen, Beijing, China). As an internal reference, the expression levels of the housekeeping gene GADPH were employed. The amplification program was according to the methods of [31], and gene expression was calculated using the 2^−ΔΔCt^ method.

### 2.7. Statistical Analysis

Data are expressed as the mean ± standard deviation of three biological replicate samples. Statistical and significance analyses were performed using the data analysis software GraphPad Prism 9.2. Data variability was analyzed using two-way analysis of variance (ANOVA). Differences were considered significant when the *p* values were below 0.05. 

## 3. Results

### 3.1. Identification and Comparative Analysis of the rtt109 Gene in M. purpureus 

**Figure 1:**
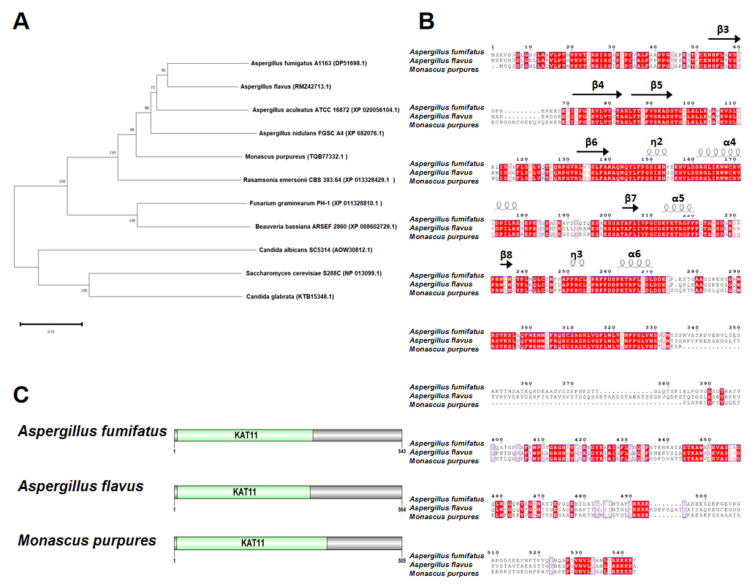
Sequence analysis of *rtt109*. (**A**): Phylogenetic trees analysis of *rtt109* orthologues in other fungal species. (**B**): Analysis of the conserved domains of *rtt109* in *Aspergillus fumifatus*, *Aspergillus flavus*, and *Monascus purpures*. (**C**): Protein domain analysis of *rtt109* in *Aspergillus fumifatus*, *Aspergillus flavus*, and *Monascus purpures*.

HAT Rtt109 homology in *M. purpureus* was identified using BLASTP, with *A. fumigatus* Rtt109 (AFUB_057090, GenBank: EDP51698.1) as the query. The DNA sequence of *M. purpureus* Rtt109 was 1518 bp, without introns encoding a 505 amino acid putative histone acetylase. Phylogenetic tree analysis revealed that *M. purpureus* Rtt109 was highly homologous to Rtt109 proteins in other fungi (Figure 1A). The comparative amino acid sequence revealed that Rtt109, in *M. purpureus*, *A. fumigatus* A1163 (GenBank: EDP51698.1), and *A. flavus* (GenBank: RMZ42713.1), had high homology (Figure 1B), and contained the same KAT11 domain at the N-terminus (Figure 1C).

### 3.2. Validation of Rtt109 Knockout Strain Δrtt109 and Complementary Strain Δrtt109:Com

**Figure 2:**
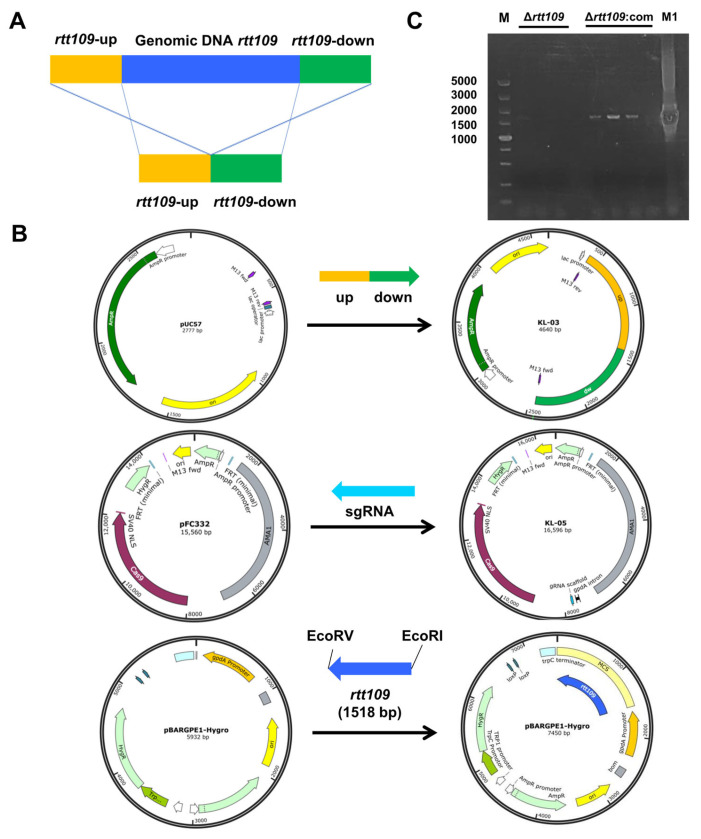
Construction of recombinant plasmid. (**A**): The deletion principle of displacement type target vector pUC57. (**B**): The KL-05 plasmid was constructed by the *rtt109* replacement gene with pUC57 vector; The KL-05 plasmid was constructed with sgRNA gene and FC332 vector; The KL-03 and KL-05 plasmid were used to construct the *rtt109* gene deletion strain. (**C**): Diagnostic PCR results of Δ*rtt109* mutants and Δ*rtt109*:com mutants. The rtt-up/rtt-dw are primers of PCR verifications designed. Lane 1 was the DL5000 DNA marker; Lines 2–4 were the PCR production of Δ*rtt109* mutants; Lines 6–8 were the PCR production of Δ*rtt109*:com; Line 9 was negative control; Line 10 was the PCR production of WT M1.

To evaluate the function of the *rtt109* gene in *M. purpureus*, a full-length deletion strain of the *rtt109* gene was constructed through homologous gene replacement, through CRISPR/Cas9. The targeting fragment was constructed using overlap PCR, with two DNA fragments amplified from *M. purpureus* genomic DNA, each containing a homology arm (Figure 2A). The KL-03(*rtt109*) and KL-05(*rtt109*) plasmids for *rtt109* gene deletion were constructed by inserting the targeting fragment, or sgRNA fragment, into pUC57 and pFC332 plasmids, respectively (Figure 2B). The overexpression plasmid pBARGEP-*hygro*-*rtt109* was constructed by inserting a *rtt109* gene fragment into the plasmid pBARGEP-*hygro* (Figure 2B). The deletion and complementation of the *rtt109* gene were verified by diagnostic PCR. As shown in Figure 2C, the *rtt109* band was absent in three colonies of the Δ*rtt109* strain, whereas the band was restored in three colonies of the complementary strain Δ*rtt109*:com (Figure 2C). 

### 3.3. HAT Rtt109 Played an Important Role in M. purpureus Growth and Development

**Figure 3:**
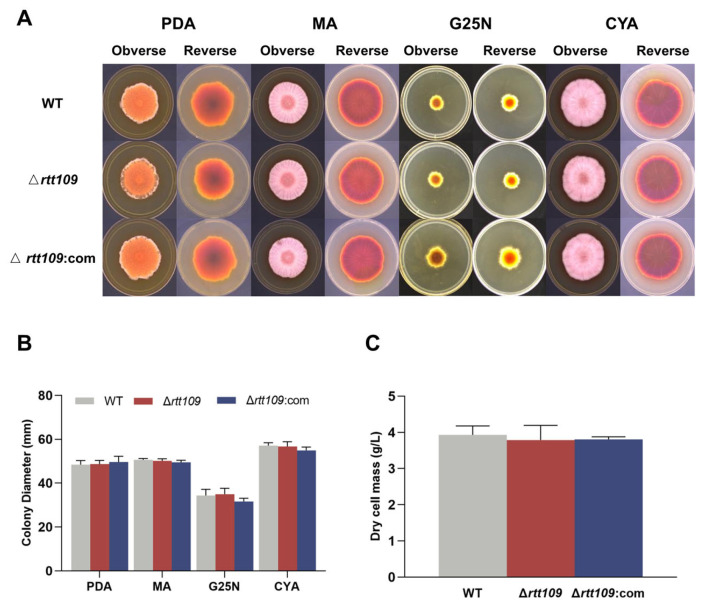
The growth and development of WT, Δ*rtt109*, and Δ*rtt109*:com strain. (**A**): The growth of WT, Δ*rtt109*, and Δ*rtt109*:com on PDA, MA, and G25N solid medium. (**B**): Colony diameter. (**C**): Biomass (dry cell mass).

To assess the effect of HAT Rtt109 on *Monascus* growth, the conidia of the corresponding strains were inoculated onto solid PDA, MA, G25N, and CYA media, respectively, at 30 °C for seven days. The colony morphology of the Δ*rtt109* strain was very similar to that of the WT strain (Figure 3A), and the colony diameter of the Δ*rtt109* strain exhibited no significant difference, compared with that of the WT strain (Figure 3B). Additionally, there was no difference in biomass between the Δ*rtt109* and the WT strains (Figure 3C).

**Figure 4:**
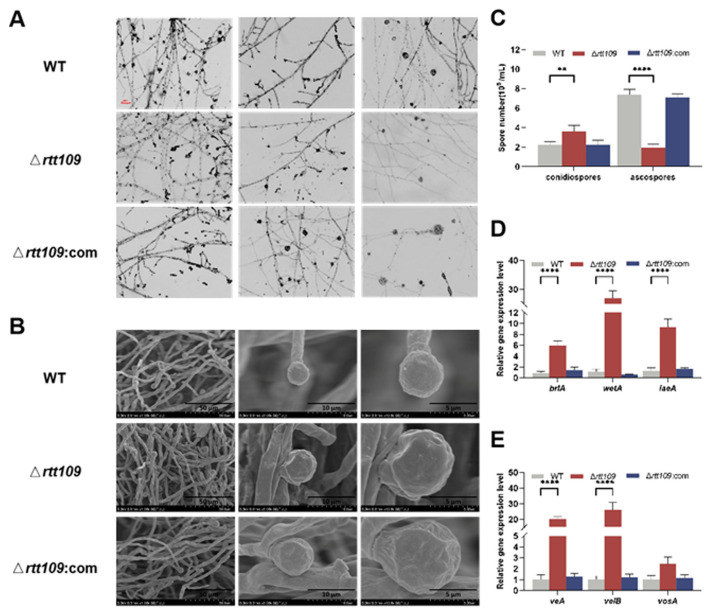
Morphology comparison of the WT, Δ*rtt109*, and Δ*rtt109*:com strain. (**A**): Microscopic observation of strains on PDA (40× microscope); Scale bar was 100 μm. (**B**): Microscopic structure of spore grown by SEM. (**C**): Comparison of spore number of ascospore and conidia. (**D**,**E**): The relative expression level of growth and conidial development regulatory genes. Two-way ANOVA and multiple comparison test were used for statistical analysis, ** *p* < 0.01 and **** *p* < 0.0001.

To obtain more detailed information on sporulation, the strains were inoculated on PDA at 30 °C for seven days. Microscopic observations showed that the Δ*rtt109* strain exhibited normal asexual development and lossy ascospore development, compared to the WT strain, but the complementary strain Δ*rtt109*:com was similar to the WT strain (Figure 4A). Scanning electron microscopy revealed that the mycelia of the Δ*rtt109* strain were curled, twisted, fragmented, and had abnormal bursa closure, whereas the complementary and WT strains showed much smoother and round mycelia (Figure 4B). In Δ*rtt109* strain, lack of *rtt109* caused few, or no, typical closed ascospores. However, many closed ascospores were observed in the WT and complementary strains. The conidiospore yield of the Δ*rtt109* strain was 1.63-fold higher than that of the WT strain (Figure 4C). The ascospore yield of the Δ*rtt109* strain was only 0.73-fold higher than that of the wild-type strain (Figure 4C). RT-qPCR was performed to analyze the expression of growth and conidial development regulatory genes, and the results showed that, in the early stage of conidia formation, the transcription levels of *brlA*, *wetA, laeA*, *veA*, and *velB* in the Δ*rtt109* strain were significantly higher than those in the WT strain, with changes of 6.93-, 24.50-, 7.08-, 19.21-, and 25.22-fold, respectively (Figure 4D,E). Similarly, the expression levels of the corresponding genes in the complementary strain Δ*rtt109*:com were very close to those in the WT strain (Figure 4D,E). 

### 3.4. The Rtt109 Gene Affects the Synthesis of MPs and CTN 

**Figure 5:**
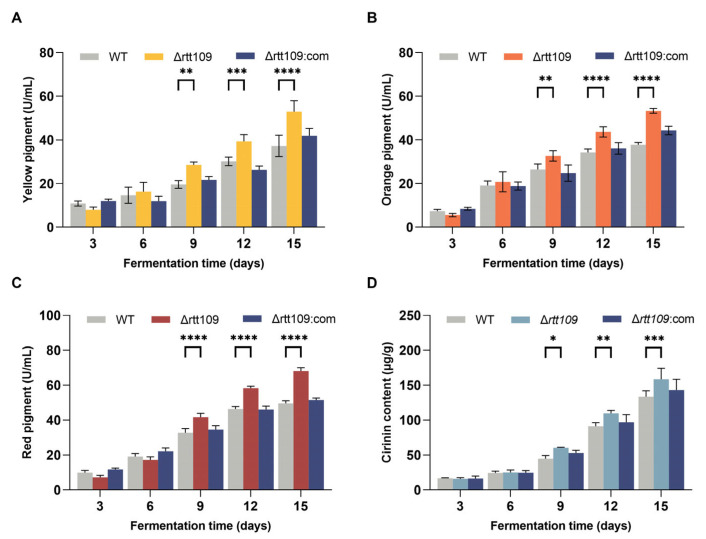
Production of MPs and CTN in the WT, Δ*rtt109*, and Δ*rtt109*:com strain. (**A**): Yellow pigment. (**B**): Orange pigment. (**C**): Red pigment. (**D**): CTN. Two-way ANOVA and multiple comparison test were used for statistical analysis, * *p* < 0.05, ** *p* < 0.01, *** *p* < 0.001, and **** *p* < 0.0001.

MPs are a mixture that can be divided into red (505 nm), yellow (410 nm), and orange (448 nm), according to the difference in maximum absorbance. MPs are the main secondary *Monascus* metabolites, and the lack of *rtt109* darkened the color of the colony (Figure 5A). Therefore, we further analyzed the content of the three pigments in the WT, Δ*rtt109*, and Δ*rtt109*:com strains grown in fermentation broth.

As shown in Figure 5, the yields of the three pigments showed almost the same trend (Figure 5A–C). The MP production in the Δ*rtt109* strain was higher than that in the WT and Δ*rtt109*:com strains. Especially on day 15, the yield of red pigments in the Δ*rtt109* strain was significantly (*p* < 0.0001) increased by 37.43%, compared with that of the WT strain. The yield of yellow and orange pigments in the Δ*rtt109* strain was significantly (*p* < 0.0001) higher on day 15 than that in the WT strain by 41.20% and 42.32%, respectively. As expected, the complementary strain Δ*rtt109*:com restored the production of the three pigments to the level observed in the WT strain (Figure 5A–C).

The CTN content in the fermentation medium of the corresponding strains peaked on day 12 (Figure 5D). WT, Δ*rtt109*, and Δ*rtt109*:com strains produced 91.23, 122.75, and 96.90 μg/g CTN, respectively (Figure 5D). The CTN production in the Δ*rtt109* strain was 34.54% higher than that of the WT (*p* < 0.001), whereas restoration of the *rtt109* gene reduced the CTN yield to the level of the WT strain.

**Figure 6:**
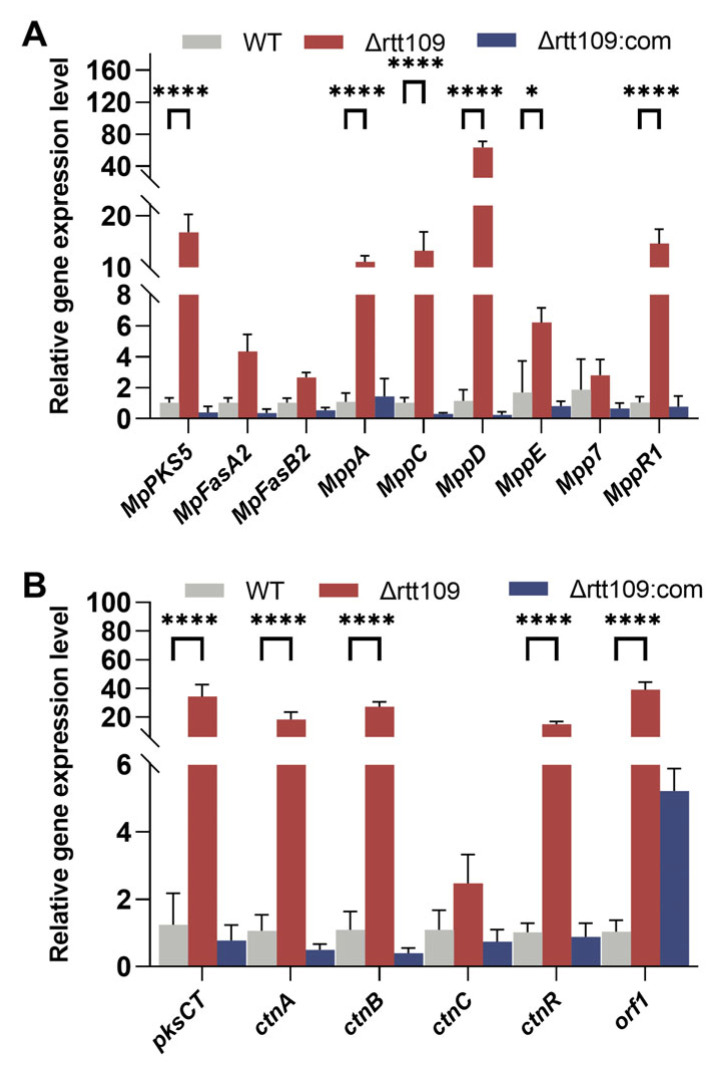
Analysis of MPs and CTN biosynthetic gene clusters in the WT, Δ*rtt109*, and Δ*rtt109*:com strain. (**A**): The relative expression level of key genes related to the biosynthetic pathway of pigments. (**B**): The relative expression level of key genes related to the biosynthetic pathway of CTN. Two-way ANOVA and multiple comparison test were used for statistical analysis, * *p* < 0.05, **** *p* < 0.0001.

We further analyzed the changes in the expression levels of genes involved in the pigment and CTN biosynthetic pathways. Deletion of *rtt109* resulted in a considerable upregulation in the expression levels of four genes (*p* < 0.0001): *MpPKS5*, *mppA, mppD*, and *mppR1*, which were 9.76-, 46.18, and 14.11 times higher, respectively, than those of the WT strain (Figure 6A). Similar to the key genes involved in MP biosynthesis, the expression of key genes related to CTN biosynthesis showed an upward trend in the *rtt109* knockout strain. As shown in Figure 6B, the relative expression levels of six genes (*pksCT*, *ctnA*, *ctnR*, *ctnB*, *ctnC*, and *orf1*) were upregulated in the Δ*rtt109* strain, and were 32.81, 21.48, 28.40, 20.59, 60.38, and 3.49 times higher, respectively, than those in the WT strain. The relative expression levels of *pksCT*, *ctnA*, *ctnR*, *ctnB*, and *ctnC* were significantly (*p* < 0.0001) upregulated in the Δ*rtt109* strain, compared to those of the WT strain. However, for the complementary strain Δ*rtt109*:com, the relative expression levels of almost all the corresponding genes showed a difference, compared with the WT strain, except for gene *orf1*.

## 4. Discussion

Histone acetylation levels, maintained by the coordinated activity of HATs and HDACs, govern chromatin shape and gene expression. It is generally accepted that HATs and HDACs play regulatory roles by altering gene expression. Histone acetylation is critical in fungi for controlling their development and pathogenicity [40]. For example, in yeast, H3K56 acetylation (H3K56ac) is present in the produced histone H3, and is required for proper ribosome assembly and genome stability. Rtt109 is a crucial HAT that regulates H3K56ac levels in yeast [17]. In the present study, we constructed *Monascus rtt109* deletion strain Δ*rtt109* and complementary strain Δ*rtt109*:com, and evaluated the effect of Rtt109 on growth, morphological development, and secondary metabolite biosynthesis in *Monascus*. 

Conidial development is an important marker of asexual reproduction in filamentous fungi. In this study, we found that *rtt109* had little effect on *Monascus* growth. The number of conidia increased 1.63-fold, and sexually reproducing spores (ascospores) decreased 0.73-fold in the Δ*rtt109* strain, compared with that in the WT strain. The complementary strain Δ*rtt109*:com behaved similarly to the wild-type strain. These results indicate that *rtt109* impacted *Monascus* spore formation. Rtt109 may balance the asexual and sexual development in *Monascus,* which is consistent with previous reports that the deletion of *rtt109* significantly affects conidial development [13,21,22,41,42,43,44]. After the loss of *rtt109*, vegetative growth was significantly inhibited in *A. flavus*. The nuclear growth of the ∆*rtt109* strain was smaller and significantly reduced in number. The number of conidia in the ∆r*tt109* strain was significantly reduced, particularly in the yeast extract sucrose solid medium [21]. In *A. fumigatus*, the Δ*rtt109* strain also showed significantly reduced conidia formation and colony growth [22]. 

As in the asexual development model of *A. nidulans*, the regulatory factors involved in spore development are roughly classified into central regulatory, upstream activating, negative regulatory, velvet regulatory, and light response regulatory factors [45]. The central regulatory network for asexual reproduction and morphological development is mainly composed of *brlA*, *abaA*, and *wetA* cascades, which coordinate gene expression and activation during conidial formation [46,47]. The central regulatory cascade consists of BrlA, AbaA, and WetA, which regulate the activation and expression of several genes during conidial formation [47]. 

In this study, Rtt109 was found to play an important role in the regulation of mycelial growth and conidia in *Monascus*, and conidial formation was significantly affected in the Δ*rtt109* strain. Therefore, we evaluated, through RT-qPCR, how deletion of *rtt109* affected the transcriptional expression of *brlA* and *wetA* (central regulatory factors), *laeA* (central regulatory factors), and *veA*/*velB*/*vosA* (velvet regulatory factors). We found that deletion of *rtt109* in *M. purpureus* resulted in a dramatic increase in the expression of velvet regulatory factors *veA/velB*. The expression of *brlA*, *wetA*, and the global regulator *laeA* was positively regulated. In particular, the *wetA* gene was upregulated 24.50-fold, which was consistent with the differentiation defects observed in the Δ*rtt109* strain. In *A. fumigatus*, the transcription levels of *wetA*, *brlA*, and *abaA,* which are regulatory genes for conidial development, decreased significantly in the *Aspergillus* Δ*rtt109* strain [22]. At the early stage of conidial development, the transcription level of *brlA* in the Δ*rtt109* strain was significantly lower than that in the WT strain in *A. flavus*. Studies have found that the meristem morphology of *Monascus* may differ from that of *Aspergillus*. The *Monascus* genome contains *brlA* and *wetA* homologs, but no *abaA* homologs [48]. Research indicates that the deletion or overexpression of *brlA* and *wetA* in *M. ruber* had no significant effect on hyphal formation in the fungus [39]. It has been speculated that the central regulation in *Aspergillus* differs from that in *Monascus*. Rtt109 activates the central regulatory genes of the asexual developmental process in *Aspergillus*; however, the differences between the asexual developmental patterns of *Monascus* and *Aspergillus* need to be further investigated. 

Pigments and CTN are the main secondary *Monascus* metabolites synthesized through the polyketide pathway [24]. CTN is a secondary metabolite produced by many fungal strains, which presents genotoxicity, carcinogenicity, and teratogenicity, and causes serious damage to human kidneys [25]. The method for restraining or eliminating CTN is a key problem in the development and industrial applications of *Monascus*. We evaluated the effect of Rtt109 on the ability of *M. purpureus* to produce pigments and CTN. The production of pigments and CTN in the Δ*rtt109* strain was 37.43–42.32%, 34.54% higher than that of the WT strain during liquid fermentation, whereas Δ*rtt109*:com strains recovered their ability to produce CTN to the same level as WT strains (Figure 5D). These results indicated that Rtt109 may regulate polyketide synthesis.

Knockout of *rtt109* affected the yield of MPs and CTN in *Monascus*. The transcriptional levels of CTN-related synthesis genes were significantly upregulated in the f*rtt109* strain. Rtt109 regulated CTN synthesis genes, which may be the reason affecting CTN yields. The pigment biosynthesis gene cluster contains the naphthoquinone biosynthesis pathway, and it is difficult to determine how Rtt109 regulates pigment biosynthesis genes. The gene expression level of the pigment biosynthesis pathway is fluctuating, which is consistent with the previous research results of HAT MrGcn5 [27]. The CTN production of *Monascus* fermented by the *MrGcn5* deletion strain was lower than that of WT, the CTN production capacity of the *MrGcn5* supplementation strain recovered to the level of the WT. *MrGcn5* regulates the production of CTN and expression of key genes involved in CTN synthesis in *M. ruber*. Similar to the regulation of CTN by histone deacetylase *MrRpd3* in *Monascus*, overexpression of *Mrrpd3* had little effect on *Monascus* azaphilone pigments, but significantly increased the CTN content in *M. ruber* [28]. Different HATs have different regulatory characteristics in *Monascus*, including effects on spore development and secondary metabolites.

In this study, we demonstrated that the histone acetyltransferase Rtt109 is involved in the regulation of several physiological processes in *Monascus*. Knocking out the *rtt109* gene affected the growth and conidia development in *Monascus*, synthesis of pigments and citrinin, and transcriptional expression of key genes. The role of Rtt109 in *Monascus* was reported for the first time, thereby enhancing our current knowledge of the development and regulation of secondary metabolism in the fungus. In addition, this study revealed various mechanisms that regulate citrinin production.

## Data Availability

Data will be made available on request.

## Supplementary Materials

The following supporting information can be downloaded at: https://www.mdpi.com/article/10.3390/jof9050530/s1, Table S1. Primer sequences used in this study. Table S2. Primer sequences for the key genes in *Monascus purpureus* M1.

## Abbreviations

HAT	histone acetyltransferase
HDAC	histone deacetylase
H3K56	histone 3 lysine 56
MP	*Monascus* pigment
MK	monacolin K
GABA	γ-aminobutyric acid
CTN	citrinin
NCBI	National Center for Biotechnology Information resource
WT	wild-type
PDA	potato dextrose agar
MA	malt extract agar
G25N	25% nitroglycerin agar
CYA	chapek yeast extract agar
PDB	potato dextrose broth
ELISA	enzyme-linked immuno sorbent assay
SEM	scanning electron microscope
PBS	Phosphate-saline
HMDS	hexamethyldiazolidine
RT-qPCR	real-time quantitative PCR
ANOVA	analysis of variance
