# Supplementary material for: Histone Acetyltransferase Rtt109 Regulates Development, Morphogenesis, and Citrinin Biosynthesis in Monascus purpureus
Source: J Fungi (Basel). 2023 Apr 29;9(5):530. doi: 10.3390/jof9050530 (PMC10219144; doi:10.3390/jof9050530)
Supplement: Supplementary file 1 [file jof-09-00530-s001.zip › jof-2295681-supplementary.pdf]

Supplementary Material

Histone acetyltransferase Rtt109 regulates development, morphogenesis, and citrinin biosynthesis in *Monascus purpureus*

Ruoyu Shi [1,2,†], Pengfei Gong [1,†], Qiaoqiao Luo [1], Wei Chen [1,*] and Chengtao Wang [1,*]

[1]Beijing Advanced Innovation Center for Food Nutrition and Human Health, Beijing Engineering and Technology Research Center of Food Additives, Beijing Technology & Business University (BTBU), Beijing, 100048, China

[2]Yunnan Plateau Characteristic Agricultural Industry Research Institute, Yunnan Agricultural University, Kunming, 650201, China

[*]Correspondence: weichen@btbu.edu.cn (W.C.); wangchengtao@th.btbu.edu.cn (C.T. W)

Tel.: +86-10-68984003 (Wei Chen); +86-10-68984003 (Chengtao Wang)

[†]These authors contributed equally to this work.

Table of Contents

Table S1. Primer sequences used in this study.

Table S2. Primer sequences for the key genes in *Monascus purpureus* M1.

Table S1. Primer sequences used in this study.

Primers	Primer sequences	Application
rtt-up-FOR	accatgattacgccaagcttctcttcgaggacgttgaccg	To amplify upstream fragment of target gene *rtt109*.
rtt-up-REV	cgagaaatatccaacgcctgctgaccttggcaaatggcgt	
rtt-dw-FOR	acgccatttgccaaggtcagcaggcgttggatatttctcg	To amplify downstream fragment of target gene *rtt109*.
rtt-dw-REV	aaacgacggccagtgaattctcggtcccatgggataatct	
pUC57-kortt-FOR	aagcttggcgtaatcatggtcatagctgtttcctg	The pUC57 homologous main chain and upstream and downstream fragments were amplified to produce pKL-03.
pUC57-kortt-REV	gaattcactggccgtcgttttacaacgtcgtgac	
pFC322-kortt-sgRNA-DNA1-FOR	cagtcatctgcggaacatatactgggcccgggaagatctgcgtaagctccctaattggc	Primers of piece 1 for construction of pKL-05. The bold 6 bp-oligonucleotides is reverse complementing bases.
pFC322-kortt-sgRNA-DNA1-REV	gacgagcttactcgtttcgtcctcacggactcatcagTCAAATcggtgatgtctgctcaagc	
pFC322-kortt-sgRNA-DNA2-FOR	gatgagtccgtgaggacgaaacgagtaagctcgtcTCAAATCTTTGGATCAGTTCgttttagagctagaaatag	Primers of piece 2 for construction of pKL-05. The underlined 20 bp-oligonucleotides is the sgRNA sequence
pFC322-kortt-sgRNA-DNA2-REV	gggcggtgattctgctgtctcggctgaggtcttaattaagagccaagagcggattcctc	

Table S2. Primer sequences for the key genes in *Monascus purpureus* M1.

Genes	Primer sequences (5′to3′)	Length (bp)	Tm Value	Description
brIA F	ATGTCAGGGTGGCGTGAAGT	20	60	asexual developmen
brIA R	CCTGAACTGTACCTGCTTGAT	21	56	
wetA F	ATGTGTTATATTCCCCGGGA	20	60	asexual development
wetA R	TTAGCAGAGTGCGGCCTCGAG	21	62	
laeA F	ACTCGTAGCGGATGTAAGA	19	55	global regulator
laeA R	CCGTGCTTGGTAGATGTG	18	55	
velvet1 F	CCCGTGTTGACTGGTGTT	18	55	velvet regulatory factors
velvet1 R	AGCCCAGGAAACTTCTTGGCACTAT	25	61	
velvet2 F	CCTCCGTGTATCCGTTTG	18	61	velvet regulatory factors
velvet2 R	TTGCCGAGTGCTTAACCAGATTCAC	25	60	
velvet3 F	CAGACCAGGGTGTCAAGTT	19	54	velvet regulatory factors
velvet3 R	GTTTCGTGCTCCATAATCC	19	54	
MpPKS5 F	TGTCCGACGAGTTTCTGCAA	20	58	NR-PKS
MpPKS5 R	TATCAACGCTGCTTGGGCAT	20	60	
MpFasA2 F	ATGGATCGCCCGATCTTGTC	20	59	FAS subunit alpha
MpFasA2 R	CTTTGTCGAGTCCGCTGGAT	20	59	
MpFasB2 F	CCTCCAGGGATTACAACCCG	20	58	FAS subunit beta
MpFasB2 R	ATTCAATGCCAGGTGCTCCA	20	58	
mppA F	TCCCGTTTCTTGGACGTGAG	20	59	C-11-ketoreductase
mppA R	ACGTGCCATGGTTCTGTCTT	20	59	

mppB F	CGTCTCGCCCGATAACTTCA	20	60	acyltransferase
mppB R	TTGACAGACGGGTCGAAGTC	20	58	
mppC F	CAGTCCTCGTCCCTTCCAGT	20	60	NADPH-dependent oxidoreductase
mppC R	CCACGGTGAAGGATGTCGAG	20	58	
mppD F	TCAACACGGGAGATGCTGTC	20	62	serine hydrolase
mppD R	GCCAAAGGACAGGAGCAGAT	20	63	
mppE F	CTTCCCGATGCCGTTGTGAT	20	60	enoyl reductase
mppE R	GTCTCGTGGATCATCTCGT	19	60	
mppR1 F	TCTGCAGTATGCCATGTGGG	20	59	transcription factor
mppR1 R	ATGGCACCGTCACTTAGCTC	20	55	
mppR2 F	ACGAAACCCTCCATGACACC	20	59	transcription factor
mppR2 R	TGCAGACAGCCTTGTGGTAG	20	59	
pksCT F	GTCGAACTTGGAATCGATTCGCTG	24	60	Polyketide synthase
pksCT R	TCACTTCCGTAAAGGCACGGTAG	24	59	
ctnA F	GACGTCAAATCACGCTGCAGG	21	61	citrinin biosynthesis oxygenase
ctnA R	GTCCGTATGCGCACTACTGCC	21	62	
ctnB F	ATGAAAGGGCAGACAGGG	18	54	citrinin biosynthesis oxydoreductase
ctnB R	GGAAGATGGCAGCATTAGA	19	52	
ctnC F	CCTTCCCGAGACATACCA	18	53	citrinin biosynthesis transporter
ctnC R	AAGAAGAGGTATAAGATTCCATAGA	25	51	
ctnR F	GGTGCAGGCAGAATTAAACCAACTG	25	59	transcriptional activator
ctnR R	GGCATTTCTGGCCCAGCCTAG	21	60	

orf1 1F	CGTCCCTCCTACGCTGTT	18	56	dehydrogenase
orf1 1R	GCTTCCTCCTCCGTTTCA	18	54	
GAPDH F	CCGTATTGTCTTCCGTAAC	19	55	Reference gene
GAPDH R	GTGGGTGCTGTCATACTTG	19	56	
